# Editorial: Exploring herbal innovations: new frontiers in functional foods and nutraceuticals development

**DOI:** 10.3389/fphar.2026.1889707

**Published:** 2026-07-07

**Authors:** Xuejing Zhong, Meiyu Song, Hanping Bao, Minhui Li, Chunhong Zhang

**Affiliations:** 1 School of Pharmacy, Baotou Medical College, Baotou, China; 2 Inner Mongolia Key Laboratory of Characteristic Geoherbs Resources Protection and Utilization, Baotou Medical College, Baotou, China; 3 Institute of Traditional Chinese Medicine, Inner Mongolia Traditional Chinese and Mongolian Medical Research Institute, Hohhot, China

**Keywords:** functional foods and nutraceuticals, health modulation by natural products, herbal bioactive discovery, medicinal and health food plants and fungi (MHFPs), translational nutrition

Medicinal and health food plants and fungi (MHFPs) are gaining traction in the realm of functional foods and nutraceuticals by merging traditional medicinal approaches with modern dietary innovations. These natural sources are valued for their nutritional and therapeutic properties, which are essential for creating health-enhancing products. As consumer demand for natural and effective health solutions continues to grow, MHFPs based functional foods are increasingly being recognized as key components of sustainable health practices. Centered on the core theme of “optimizing MHFPs to develop effective and sustainable functional foods and nutraceuticals,” [SIC] this Research Topic compiled eight studies that provide multi-level scientific evidence—from chemical characterization and mechanistic investigation to clinical evaluation—relevant to the development of MHFPs based functional foods.

At the mechanistic level, Chen et al. demonstrated in clinical specimens that patients with triple-negative breast cancer (TNBC) and high *OLFML2A* expression exhibit reduced *E-cadherin* and elevated *N-cadherin*, *Vimentin*, and *Elastin*, whereas patients with low *OLFML2A* expression showed no significant epithelial-mesenchymal transition (EMT) changes. *In vitro* experiments demonstrated that konjac (*Amorphophallus konjac* K. Koch) petroleum ether extract inhibits the migration and invasion of TNBC cells in a dose-dependent manner and that these effects are associated with the downregulation of *OLFML2A* expression and reversal of EMT. Lee et al. systematically validated the triple protective mechanism of the ethyl acetate fraction of *Boehmeria nivea* (L.) Gaudich. leaves (EA-BN) in a chronic obstructive pulmonary disease (COPD) model that involved the activation of the Nrf2 antioxidant pathway and the inhibition of thioredoxin interacting protein (TXNIP)/NLRP3 inflammasome and apoptotic signaling; transcriptomic analysis further expanded the understanding of its molecular network.

In the study of intestinal protection, Yin et al. systematically characterized the structure of Bletilla oligosaccharides (BO) and confirmed that BO can alleviate chemotherapeutic intestinal mucositis. This mechanism involves attenuating inflammatory responses via the TLR4-NF-κB/MyD88 signaling pathway, protecting the intestinal mucosal barrier, and improving the gut microbiota community, suggesting that BO holds promise as a novel dietary supplement for the prevention and treatment of chemotherapeutic intestinal mucositis. Li et al. confirmed that Huoxiang Zhengqi dropping pills (HXZQD) maintain intestinal homeostasis under exertional heat stroke (EHS) by regulating the MAPK/NF-κB signaling pathways to preserve intestinal barrier function and improve gut microbiota composition.

Notably, three clinical studies provided high-level evidence for the evidence-based evaluation of MHFPs. Of these, Ciappina et al. suggested that integrative mycotherapy may represent a feasible supportive approach for selected patients experiencing treatment-related hematologic toxicity. Furthermore, Lee et al. demonstrated in a randomized controlled trial that Bojungikgi-tang (BJT) improves anorexia and atopic dermatitis symptoms and that these effects are potentially associated with modifications in the gut microbiota. Zhang et al. showed in a randomized, double-blind, placebo-controlled trial that TiaoPi AnChang Decoction (TPACD) reduces CAPOX-induced adverse reactions and improves quality of life in patients with colorectal cancer. Jha et al. systematically reviewed the supportive therapeutic potential of plant metabolites in metastatic breast cancer and the advances in nanodelivery technologies, noting that the vast majority of evidence remains at the preclinical stage and that the lack of bioavailability and clinical validation continues to be the primary barrier to translation.

Overall, the studies compiled in this Research Topic illustrate the multi-level scientific value and application potential of MHFPs in the development of functional foods and nutraceuticals, especially for addressing health concerns such as chronic inflammation, chemotherapy-induced adverse reactions, and multiple organ injury. From the targeted regulation of plant active metabolites and multi-pathway modulation of classical herbal formulas to the restoration of gut microbiota homeostasis and modulation of host immunity and oxidative stress response, along with the alleviation of treatment-related toxicities, these studies have systematically uncovered the pharmacological effects of MHFPs against chemotherapy-induced intestinal mucositis, COPD, and atopic dermatitis, in addition to their value in supportive tumor therapy. Nevertheless, despite the remarkable progress achieved in phases, numerous obstacles hinder the translation of basic functional food research into clinical nutritional intervention. For instance, several studies are limited to animal experiments and lack human clinical trials, and existing clinical trials feature small sample sizes, restricting the extrapolation of research outcomes. The majority of mechanistic investigations only center on single signaling pathways, lacking systematic exploration of upstream and downstream regulatory networks and alternative bypass pathways. Research concerning the modulatory effects on gut microbiota mostly remains at the level of compositional description without functional validation. Pharmacokinetic and toxicological data are also insufficient, leaving the *in vivo* absorption, distribution, metabolism, and long-term safety of active metabolites undefined. In addition, imperfect chemical characterization approaches and incomplete dosage standardization systems for extracts impair experimental repeatability and product quality control. Collectively, these Research Topics form the core bottlenecks restricting the clinical translation of functional foods and nutritional intervention strategies.

Future research should conduct active monomeric metabolite isolation and structure-activity relationship analysis along with unified raw material chemical characterization for various MHFPs crude extracts; in addition, it should improve *in vitro* cellular assays and *in vivo* experiments using multi-strain animals and clinically relevant pathological models. Multi-omics, network pharmacology, and fecal microbiota transplantation technologies should be adopted to elucidate signaling pathways and gut microbiota-associated mechanisms more deeply, distinguishing specific pharmacological activities from non-specific cytotoxicity. Meanwhile, pharmacokinetic and short- and long-term toxicological assessments should be implemented, and nano-delivery technologies should be utilized to enhance the bioavailability of active metabolites. Large-sample, randomized, double-blind, and placebo-controlled designs are recommended for clinical trials, with subject stratification based on disease molecular subtypes and traditional Chinese medicine syndrome differentiation alongside extended follow-up periods. These designs will be able to explore the synergistic toxicity-reducing effects of MHFPs combined with chemoradiotherapy and facilitate the translation from preclinical research to the individualized clinical application of MHFPs derived preparations.

In summary, these studies underscore the necessity of integrating MHFPs related research with chemical characterization techniques, gut microbiota analysis, and clinical evaluation methods. By systematically addressing translational barriers and prioritizing reproducibility, standardization, and clinical validation, the field can advance toward the development of safe, effective, and scalable functional foods and nutritional interventions, thereby addressing health challenges such as chronic diseases and chemotherapy-induced adverse reactions. The editorial team sincerely thanks all the authors and reviewers for their valuable contributions to this Research Topic.

